# Effect of stroke location on the laryngeal cough reflex and pneumonia risk

**DOI:** 10.1186/1745-9974-1-4

**Published:** 2005-08-04

**Authors:** W Robert Addington, Robert E Stephens, John G Widdicombe, Kamel Rekab

**Affiliations:** 1 Brevard Rehabilitation Medicine, 200 Ocean Avenue, Suite 201; Melbourne Beach, Florida, 32951, USA; 2 Chair, Department of Anatomy, Kansas City University of Medicine and Biosciences, 1750 Independence Avenue, Kansas City, Missouri, 64106, USA; 3 Emeritus Professor, University of London, 116 Pepys Road, London SW20 8NY, UK; 4 Chair, Department of Mathematics and Statistics, University of Missouri–Kansas City; Kansas City, Missouri, USA

**Keywords:** cough, reflex, laryngeal, stroke, pneumonia, brainstem shock

## Abstract

**Background:**

The purpose of this study was to evaluate the risk of developing pneumonia in acute stroke patients comparing the early anatomical stroke location and laryngeal cough reflex (LCR) testing.

**Methods:**

A prospective study of 818 consecutive acute stroke patients utilizing a reflex cough test (RCT), which assesses the neurological status of the LCR compared to magnetic resonance imaging or computerized tomography for stroke location and subsequent pneumonia outcome. Stroke diagnosis and stroke location were made by a neurologist and clinical radiologist, respectively; both were blinded to the RCT results.

**Results:**

Brainstem (p-value < .007) and cerebral strokes (p-value < .005) correlated with the RCT results and pneumonia outcome. Of the 818 patients, 35 (4.3%) developed pneumonia. Of the 736 (90%) patients who had a normal RCT, 26 (3.5%) developed pneumonia, and of the 82 (10%) patients with an abnormal RCT, 9 (11%) developed pneumonia despite preventive interventions (p-value < .005). The RCT had no serious adverse events.

**Conclusion:**

The RCT acted as a reflex hammer or percussor of the LCR and neurological airway protection and indicated pneumonia risk. Despite stroke location, patients may exhibit "brainstem shock," a global neurological condition involving a transient or permanent impairment of respiratory drive, reticular activating system or LCR. Recovery of these functions may indicate emergence from brainstem shock, and help predict morbidity and mortality outcome.

## Background

The laryngeal cough reflex (LCR) protects the supraglottic larynx from significant aspiration of food or fluids during inspiration or pharyngeal spillage during swallowing [1]. The reflex cough test (RCT), using nebulized tartaric acid solution, provides an effective stimulus to the receptors in the supraglottic mucosa, and, like a reflex hammer or percussor, triggers a cascade of neurological activity in both craniospinal nerves and the central nervous system. The vagus nerve mediates the afferent component of the LCR. Tartaric acid-induced cough stimulates rapid adapting receptors (RARs) in the supraglottic region of the larynx and sensory impulses are conveyed to the medulla via the middle ramus of the internal branch of the superior laryngeal nerve (ibSLN) and vagus nerve [2-6]. The fibers of the ibSLN terminate on neurons near the nucleus tractus solitarius (NTS) of the medulla. The central connections of the reflex and voluntary cough circuits have been reviewed,[7,8] Although the central connections of reflex cough are unclear, research suggests a rapid latency [5]. Central processing of the cough reflex quickly sets off a cascade of synchronized central and peripheral responses involving the nucleus ambiguus, retroambigualis, phrenic nucleus, and medial motor cell column which project to the vagus, phrenic, intercostal and thoracoabdominal nerves, respectively [9].

Human studies have indicated the clinical implications of central and peripheral lesions of the cough system. The LCR may be impaired in individuals who have a transient (e.g., post-general anesthesia) or permanent (e.g., post-stroke, cervical cord trauma, Parkinson's disease, amyotrophic lateral sclerosis) neurological event, which may affect the afferent, central or efferent components of the LCR [10-18]. The purpose of this study was to determine the effect of identifying the initial radiological anatomical stroke location on the laryngeal cough reflex test result and the relationship to the subsequent risk of developing pneumonia.

## Methods

This was a clinical prospective study of 818 consecutive patients during a three year period of time, who were admitted to an acute rehabilitation hospital with a primary diagnosis of acute stroke. Stroke diagnosis was made by a neurologist and the stroke location was determined by a neuroradiologist, both were blinded to the RCT findings. In this study, stroke location was noted according to computer tomography (CT) or magnetic resonance imaging (MRI) results as reported by the radiologist in the clinical setting. Stroke locations were categorized as: cerebral, brainstem, multiple CNS infarcts, basal ganglion, cerebellar, or location not specified by MRI or CT report.

Upon admission to the acute rehabilitation hospital, all patients were tested with the RCT, as the first component of a standard bedside swallow examination. The RCT (Pneumoflex Systems, Inc., Melbourne Beach, FL) comprised a 20% solution of pharmaceutical grade L-tartaric acid dissolved in sterile 0.15 M NaCl solution and inhaled from a Bennett Twin Nebulizer (3012-60 ml, Puritan-Bennett Company, Carlsbad, CA). During the inhalation, the subject's nose was pinched closed. The nebulizer output was 0.2 ml/min [1,3,19-23]. The RCT was administered at bedside by a either a respiratory therapist or speech pathologist. The subject was asked to exhale, then insert the mouthpiece, and take a sharp, deep inhalation. Leakage around the mouthpiece and "puffing" the nebulizer were not considered effective inhalations. The test was administered by a either a speech pathologist or respiratory therapist at bedside and required less than five minutes to complete. The expected result of a normal RCT was an immediate series of forceful coughs, which are primarily expiratory "airway clearing" in character. A normal finding indicated a normal function of the LCR, vagus nerve, and a neurologically protected airway. If the subject had a normal RCT, additional inhalations of the RCT were not performed. The expected result of an abnormal RCT was represented by an absence of coughing, or a diminished (weak) coughing, or coughing not immediately after administration of the test stimulus. An abnormal finding indicated dysfunction of the LCR, vagus nerve or the reflex cough system, and a neurologically unprotected airway. The RCT was terminated when the subject either elicited a cough or failed to cough after three valid inhalations. The subjects were then treated clinically based on the RCT findings. The previously published RCT algorithm was followed for subsequent feeding strategies such as restricted diet, nothing by mouth (NPO) or nutritional support via percutaneous endoscopic gastrostomy (PEG) [1]. These treatment strategies were noted for all patients.

Subjects were monitored for the development of pneumonia during their hospital stay of approximately one month. Pneumonia was diagnosed as a subject having respiratory symptoms with either temperature greater than 101°, leukocytosis, or both, and an infiltrate confirmed by chest x-ray. Adverse events of the RCT were collected for all subjects.

### Data Analysis

Statistics were generated using SPSS 10.0.5. Subjects who had either a normal or abnormal RCT finding were statistically compared as to gender, age, and length of stay in the acute care setting. The principal endpoint for the study was the development of pneumonia among acute stroke patients. This endpoint is binary. An appropriate test of significance for this situation was the Fisher's exact test with the Null Hypothesis stating that among the acute stroke patients there is no significant difference in the development of pneumonia, regardless of the stroke location, between patients that had a normal RCT and those patients that had an abnormal RCT. In addition to a test of significance, it was important to determine a 95 percent confidence interval for p_1 _- p_2_, where p_1 _was the proportion of acute stroke patients that developed pneumonia and had an abnormal RCT, and p_2 _was the proportion of acute stroke patients that developed pneumonia and had a normal RCT. For completeness, the odds in favor of not developing pneumonia among the patients who had an abnormal RCT were compared to the odds in favor of not developing pneumonia among the patients who had a normal RCT. As part of a power analysis, there are standard formulae for determining sample sizes for the comparison of the proportions. The level of significance, power of the test and the proportions were evaluated. The RCT results, stroke location and pneumonia outcome were crosstabulated utilizing the Chi-Square test.

The issue of sensitivity and specificity of the RCT in determining pneumonia risk, though obviously important, is not appropriate to assess in this study because the interventions, guided by the results of the RCT, can effect pneumonia outcome [1,22,24].

## Results

The mean age of these patients was 73.69 ± 10.44, and included 426 males and 392 females. The patients in this acute stroke population included more than 59 overall comorbidies. The mean length of stay in the acute care and rehabilitation hospitals was 7.7 ± 7.7 and 31.2 ± 18.4 days, respectively. Analysis of gender, age, and length of stay in the acute care setting indicated that there were no epidemiological differences between subjects who had either a normal or abnormal RCT finding.

The principal endpoint for the study is the development of pneumonia. Among the 818 acute stroke patients, 736 (90%) patients had a normal RCT, of which 26 patients (3.5%) developed pneumonia (Table 1). Eighty-two (10%) patients had an abnormal RCT, defined as weak or absent. Of the abnormal RCT group, 69 (84%) patients had a weak RCT, of which 7 (10%) developed pneumonia. Thirteen (16%) patients had absent RCT and 2 (15%) developed pneumonia. A significant difference for pneumonia outcome was found (p-value < .005) (Table 2). The 95 percent confidence interval for p_1 _- p_2 _was (.04, .11), respectively. This two-sided 95 percent confidence interval clearly showed that p_1 _is greater than p_2_. The proportion of acute stroke patients that developed pneumonia and had an abnormal RCT was significantly greater than the proportion of acute stroke patients that developed pneumonia and had a normal RCT. In fact, 3.5% of the patients with a normal RCT versus 11% of the patients with an abnormal (weak or absent) RCT developed pneumonia.

**Table 1:** Reflex Cough Test × Pneumonia in Rehabilitation Crosstabulation

	**Pneumonia in Rehabilitation**		**Total**
**Reflex Cough Test**	Yes	No	
**Normal**	26	710	736
**Abnormal**			
**Weak**	7	62	69
**Absent**	2	11	13
**Total**	35	783	818

**Table 2:** Chi-Square Tests on the Effects of RCT on Pneumonia Outcome

	**Value**	**df**	**Asymp. Sig. (2-sided)**	**Exact Sig. (2-sided)**	**Exact Sig. (1-sided)**
Pearson Chi-Square	9.980	1	.002		
Continuity Correction	8.245	1	.004		
Likelihood Ratio	7.428	1	.006		
Fisher's Exact Test				.005	.005
Linear-by-Linear Association	9.967	1	.002		
N of Valid Cases	818				

a Computed only for a 2×2 table

b 1 cells (25.0%) have expected count less than 5. The minimum expected count is 3.51.

The odds in favor of not developing pneumonia among the patients who had an abnormal RCT were compared to the odds in favor of not developing pneumonia among the patients who had a normal RCT. The odds ratio test indicated that the odds in favor of not developing pneumonia for acute stroke patients with an abnormal RCT were significantly smaller than the odds in favor of not developing pneumonia for acute stroke patients that had a normal RCT. In fact, the ratio of the odds was .297, which was significantly smaller than 1, and a 95 percent confidence interval for the odds ratio was (.134, .658). When the level of significance is fixed at 0.05, the power of a two-sided test is 80 percent.

Stroke location, RCT results and pneumonia outcomes are shown in Table 3. Crosstabulation of the RCT results, pneumonia in rehabilitation and brainstem infarcts was significant at identifying the risk of developing pneumonia (p = .007) as was the crosstabulation for cerebral hemispheric infarcts (p = .005). Basal ganglionic, cerebellar, multiple infarcts or stroke location not specified by CT or MRI did not correlate with RCT results and predicting the development of pneumonia. Data analysis for basal ganglion and cerebellar infarcts were not crosstabulated because none of these patients developed pneumonia in rehabilitation.

**Table 3:** Stroke Location, Reflex Cough Test (RCT) Results and Pneumonia Outcome

**RCT × Pneumonia in Rehabilitation × Brainstem Infarct Crosstabulation and Chi-Square Test**			
**Reflex Cough Test**	**Pneumonia in Rehabilitation**		**Total**
		
	**Yes**	**No**	

**Normal**	1	55	56
**Abnormal**	3	6	9
**Total**	4	61	65
Fisher's Exact Test p = .007			
**RCT × Pneumonia in Rehabilitation × Cerebral Infarct Crosstabulation and Chi-Square Test**			

**Reflex Cough Test**	**Pneumonia in Rehabilitation**		Total
		
	Yes	No	

**Normal**	9	355	364
**Abnormal**	5	31	36
**Total**	14	386	400
Fisher's Exact Test p = .005			
**RCT × Pneumonia in Rehabilitation × Stroke Location Not Specified Crosstabulation and Chi-Square Test**			

**Reflex Cough Test**	**Pneumonia in Rehabilitation**		Total
		
	**Yes**	**No**	

**Normal**	13	210	223
**Abnormal**	1	27	28
**Total**	14	237	251
Fisher's Exact Test p = .522			
**RCT × Pneumonia in Rehabilitation × Multiple CNS Infarcts Crosstabulation**			

**Reflex Cough Test**	**Pneumonia in Rehabilitation**		**Total**
		
	**Yes**	**No**	

**Normal**	3	48	51
**Abnormal**	0	4	4
**Total**	3	52	55
Fisher's Exact Test p = .794			
**Reflex Cough Test × Pneumonia in Rehabilitation × Basal Ganglion Infarcts Crosstabulation**			

**Reflex Cough Test**	**Pneumonia in Rehabilitation**		**Total**
		
	**Yes**	**No**	

**Normal**	0	24	24
**Abnormal**	0	3	3
**Total**	0	27	27
**Reflex Cough Test × Pneumonia in Rehabilitation × Cerebellar Infarcts Crosstabulation**			

**Reflex Cough Test**	**Pneumonia in Rehabilitation**		**Total**
		
	**Yes**	**No**	

**Normal**	0	18	18
**Abnormal**	0	2	2
**Total**	0	20	20

Thirty-two (3.91%) of the 818 patients had a percutaneous endoscopic gastrostomy (PEG) while in rehabilitation (Table 4). Ten PEGs were placed while in rehab, and 15 PEGs were removed in rehabilitation. Seven (0.9%) of the 818 patients received a modified barium swallow (MBS) examination.

**Table 4:** Crosstabulation of Pneumonia in Rehabilitation, Reflex Cough Test, Stroke Location and PEG

Pneumonia in Rehabilitation	Reflex Cough Test	Location of Stroke	Percutaneous Endoscopic Gastrostomy		Total
				
			**Yes**	**No**	
**No**	**Normal**	Cerebral Infarct	6	349	355
		Basal ganglia	3	21	24
		Brainstem infarct	5	50	55
		Cerebellar infarct	2	16	18
		Multiple Infarctions	2	46	48
		Location not determined	1	209	210

	**Abnormal**	Cerebral Infarct	8	23	31
		Basal ganglia	1	2	3
		Brainstem infarct	1	5	6
		Cerebellar infarct	0	2	2
		Multiple Infarctions	1	3	4
		Location not determined	0	27	27

**Yes**	**Normal**	Cerebral Infarct	1	8	9
		Brainstem infarct	1	0	1
		Multiple Infarctions	0	3	3
		Location not determined	0	13	13

	**Abnormal**	Cerebral Infarct	0	5	5
		Brainstem infarct	0	3	3
		Location not determined	0	1	1

**Total**			32	786	818

Seventeen patients (2.1%) were transferred to acute care from rehabilitation, 15 had a normal RCT and 3 of these patients developed pneumonia in rehabilitation. Two of the transferred patients had an abnormal RCT and neither developed pneumonia in rehabilitation. Two of the 818 patients died in rehabilitation. One patient had a left cerebral hemispheric infarct, and died of complications secondary to cancer. The other patient had a middle cerebral artery infarct, and died four days after admission to the rehabilitation hospital due to ongoing complications secondary to pneumonia acquired in acute care.

In this study, there were no serious adverse medical sequelae from RCT administration. In the 82 stroke patients who had an abnormal RCT, there was no statistical correlation for comorbidities such as congestive heart failure, diabetes mellitus, chronic obstructive pulmonary disease, or patients who had been intubated.

## Discussion

Nebulized tartaric acid appears to be an effective, specific and safe stimulus to laryngeal receptors and testing neurological airway protection. This concentration of nebulized tartaric acid-induced cough has been used in a number of cough sensitivity studies involving normal subjects, smokers and asthmatics without causing bronchoconstriction [19-21,25,26]. Our studies on stroke subjects and other patients with neurological impairment use a single breath inhalation protocol similar to Choudry and Fuller [27]. When the internal branch of the superior laryngeal nerve was completely, bilaterally anesthetized, the LCR was transiently absent in normal subjects and they could tidal breathe nebulized tartaric acid without eliciting laryngeal or tracheobronchial cough [4]. Testing the neurological function of the LCR may help indicate those patients who are at risk of respiratory complications such as pneumonia.

This study reported a significant relationship among RCT results, pneumonia risk and both brainstem and cerebral strokes. Although all subjects had a primary diagnostic code of stroke and the initial stroke location was determined and described by a radiologist in the clinical setting, it is not always possible to determine the extent of the neurological deficits by the location of the infarct emergently using MRI or CT. This study, using the present examination techniques for identifying neurological deficits in the emergency setting showed that subjects, who had a subsequent brainstem or cerebral hemispheric infarct identified by CT or MRI and a subsequently impaired LCR, were at risk of developing pneumonia. Indeed, a clinician in the emergency room presently could not test the status of a patient's involuntary neurological airway protection, i.e., LCR, before making a decision to place a nasogastric (NG) tube or administer food, fluid or medications orally. The use of a NG tube in the acute stroke setting, without the knowledge of the neurological status of the LCR may be a significant contributing factor for the development of respiratory complications such as pneumonia, ventilator necessitation or death [28].

In the present study brainstem infarcts and cerebral hemispheric infarcts correlated with a significant risk of developing pneumonia, although basal ganglionic, cerebellar, multiple infarcts or stroke location not specified by CT or MRI did not correlate with RCT results and predicting pneumonia risk. None of the patients who had a basal ganglion and cerebellar infarct developed pneumonia in the acute rehabilitation setting. Nakagawa and coworkers reported that the incidence of pneumonia was significantly higher and the latency of swallowing response to the onset of was also longer in patients, who had either unilateral or bilateral basal ganglia infarcts than in patients with no infarct on CT [29]. However, they were studying the swallow reflex and silent aspiration during sleep in long term care patients and did not evaluate the LCR. Since the LCR and swallowing are separate neurological events, both must be evaluated separately. Although swallowing function is often assessed in hospital settings, testing the LCR is not presently performed although information as to the integrity of this vital, airway protective reflex would be helpful in patient management [1,22].

The numerous comorbidities of this acute stroke population along with the RCT results, stroke location, and pneumonia outcome data suggest the need to test all patients who might have an acute neurological impairment. The overall incidence of an abnormal RCT, in the rehabilitation setting day 4 or 5 post onset, was about 10% regardless of stroke location. The incidence of abnormal tests on acute stroke presentation in the emergency room would be higher. Patients with basal ganglionic, cerebellar, or infarct location unspecified and an abnormal RCT are not necessarily false positives, since the RCT results identified pneumonia risk and the need for appropriate intervention.

Although the central connections of the LCR are not clear in humans, the LCR probably has reciprocal connections with supratentorial areas that modulate or modify the LCR. Assuming adequate cognition and laryngeal sensorium, we are aware when the LCR is triggered by a noxious laryngeal stimulus–suggesting projections to the cerebrum. In humans a neurological interrelationship between the brainstem mediated LCR and supratentorial influences has been reported and an amygdalo-hypothalamo-reticular pathway has been suggested [30].

Cerebral hemispheric infarcts may, through mechanisms that are unclear at this time, suppress the LCR circuitry. Perhaps, moderate to large cerebral hemispheric lesions may result in neurotransmitter or neurophysiologic circuitry disruption or a downward pressure and/or mass effect secondary to cerebral edema, which could have an adverse effect upon these vital brainstem functions by interruption of descending facilitatory supratentorial pathways, as in spinal shock and general anesthesia. This condition is different from isolated brainstem lesions such alternating hemiplegias, lateral medullary syndrome, pontocerebellar angle syndrome or other bulbar lesions.

Suppression of the LCR tends to support our clinical observations that many cerebral hemispheric stroke patients, who show a transient or permanent impairment of the LCR, may have a condition we refer to as "brainstem shock." Brainstem shock may be defined as a global neurological condition involving a transient or permanent impairment of one or more of the following vital functions: the reticular activating system, respiratory drive, or the LCR. Frequently patients with large or small hemispheric strokes, bleeds or infarcts, may present unconscious with a depressed reticular activating system, or require intubation secondary to a depressed respiratory drive. Although presently not clinically tested in the emergency room or intensive care units, airway protection may also be impaired at this stage, and the initial emergency radiological examination may not give adequate information regarding the patient's neurological status. Patients in brainstem shock may recover reticular activating system function, respiratory drive, or neurological airway protection at different rates, similar to recovery from general anesthesia, and may be an important predictor of morbidity and mortality.

More detailed brain mapping of the lesion is not generally feasible, or available in the clinical setting. Such technologies might elucidate the connections between cerebral and brainstem structures associated with reflex cough, and would pose an interesting study. Although cough may be measured using more sophisticated techniques in the laboratory, such as electrophysiological or plethysmography, we feel that the method for grading cough, used in this study, is appropriate for the clinical setting as part of a comprehensive neurological examination.

## Conclusion

Although instrumented exams of the CNS using MRI or CT may be an important component of a neurological evaluation, they cannot adequately assess vital neurological functions such as respiratory centers in the reticular formation, consciousness or airway protective reflexes such as the LCR. Neurologists are familiar with the neurologically impaired patient, who has an unremarkable brain imaging study or one in which the stroke location is not specified. Although these results rarely mitigate the primary diagnosis of stroke, clinicians must still assess the status of these vital functions. Objective assessment of respiratory function and clinical evaluation of consciousness are commonly performed. However, bedside testing of the LCR is not currently available, yet its status plays an important role when the clinician must initiate a strategy for food, fluids and medications that is safe for the patient. The RCT may be helpful for identifying a change in neurological status or progressing cerebral edema in emergent stroke patients before identification is possible with imaging exams, thus assist in directing urgent care. The RCT examination may be helpful in assessing recovery of airway protection following extubation or general anesthesia and would be important further research for patient care.

The most powerful finding in this study is a normal RCT. In acute neurological patients, without confounding structural head and neck conditions that may prevent physical closure of the larynx during swallowing, a normal RCT reliably provides the opportunity to safely and aggressively approach emergency procedures such as NG tube placement, and the administration of food, fluids and medications in the acute setting. Further research needs to be done on other neuropathophysiological conditions for those patients who have an abnormal RCT. Further research on the neurological condition of brainstem shock in acute neurological conditions needs to be performed.

## Competing interests

Although none of the authors has been financially compensated for the research associated with this research, a commercial party with a financial interest in the reflex cough test may confer a financial benefit upon one or more of the authors. The reflex cough test (Pneumoflex^®^) of the laryngeal cough reflex is patented and trademarked by Pneumoflex Systems, LLC, Melbourne Beach, Florida. Pneumoflex^® ^has not been used commercially in the past or present. Pneumoflex Systems, LLC, is pursuing FDA and EU approval. Use of this technique in the health care system requires regulatory approval.

## Authors' contributions

RES and WRA conceived the study and drafted and revised the manuscript, WRA collected all subject data, JW helped draft and revise the manuscript, and KR performed the statistical analysis and wrote the appropriate section. All of the authors contributed to drafting the original and revised manuscripts, and have granted final approval of this published version.
